# Epidemiology of Pediatric Tumors in Quebec: A 17-Year Report of Cancer in Young People in the Canada Registry

**DOI:** 10.3390/curroncol31050204

**Published:** 2024-05-09

**Authors:** Caroline Bellavance, Benoit Lalonde, David Simonyan, Nada Jabado, Sebastien Perreault, Valérie Larouche

**Affiliations:** 1 Faculty of Medicine, Laval University, Quebec, QC GIV 0B3, Canada; 2 Geography Department, Laval University, Quebec, QC GIV 0B3, Canada; 3 Research Center, CHU de Quebec-Université Laval, Quebec, QC G1L 3L5, Canada; 4 Division of Hematology-Oncology, Montreal Children’s Hospital, Department of Pediatrics, McGill University, Montreal, QC H3A 0G4, Canada; 5 Division of Pediatric Neurology, Department of Neurosciences, CHU Ste-Justine, Montreal, QC H3T 1C5, Canada; 6 Department of Pediatric Hemato-Oncology, CHU de Quebec-Université Laval, Centre Mère-Enfant Soleil, Quebec, QC G1V 4G2, Canada

**Keywords:** incidence, childhood cancers, epidemiology, pediatrics, province of Quebec

## Abstract

Background. Cancer is the leading cause of disease-related death among children of more than 1 year of age. However, childhood cancer risk factors and etiology are yet to be fully understood. The goal of this study is to identify geographic variation among children and adolescents diagnosed with pediatric tumors between 2001 and 2018 in the province of Quebec. Methods. We analyzed pediatric patients less than 15 years of age from the Cancer in Young People in Canada (CYP-C) surveillance system who were diagnosed between 2001 and 2018 with cancer in the province of Quebec. The age-standardized age-adjusted incidence rates (AAIR) per 100,000 person years were calculated for all childhood cancers by cancer subgroups, Quebec Health regions, and age groups. Results. Overall, 3904 pediatric patients less than 15 years old were diagnosed with cancer in the province of Quebec in 2001–2018. The overall incidence rate (IR) in the province of Quebec was 16.14 (95%CL [15.56–16.73]) per 100,000 person years. For childhood cancers, regions that presented a higher AAIR were Chaudière-Appalaches and Capitale-Nationale with 18.2 and 17.5 per 100,000 person years, respectively. The incidence rates (IRs) in Chaudière-Appalaches (95% CI 1.0439–1.3532) and in Capitale-Nationale (95% CI 1.0124–1.2942) were statistically higher than the incidence in the province of Quebec (*p* = 0.0090 and *p* = 0.0310, respectively). When comparing the AAIR of the CNS tumor subgroup in Chaudière-Appalaches and in Capitale-Nationale, with the provincial average, we noticed a statistically higher incidence in Chaudière-Appalaches and a trend for Capitale-Nationale (*p* < 0.0001 and *p* = 0.0602, respectively). Conclusion. There is evidence of spatial clusters in Chaudière-Appalaches and Capitale-Nationale as areas for all childhood cancers. Further studies should be performed to investigate potential risk factors in these regions.

## 1. Importance of the Study

CYP-C is a national population-based registry that collects information about pediatric cancer. The information received from the previous CYP-C project by Larouche et al. showed the incidence discrepancy of central nervous system (CNS) tumors between some geographical areas in Quebec province. Our study evaluates the existence of statistically significant spatial-temporal clusters for sub-regional and regional levels in Quebec for all childhood cancers. It also provides statistics on the incidence rates (IRs) for all pediatric cancers in the population of Quebec from 2001 to 2018. We found that there is a significantly higher age-adjusted incidence of childhood cancers in the Health Regions of Chaudière-Appalaches and Capitale-Nationale. These findings suggest there might be some underlying etiologies (e.g., risk factors such as the environment and genetics) in these areas, and further research is needed. Ultimately, if we find there are underlying etiologies, this information could help public health authorities to take specific actions in these areas. 

## 2. Introduction 

Pediatric cancer occurs rarely and represents about 1% of new cancer cases per year in North America [1]. However, cancer is the leading cause of disease-related death among children of more than 1 year of age [2]. In 2020, for all cancers in children (defined as aged < 15 years), the age-standardized incidence rate (ASIR) was 156.1 and 145.2 per 1,000,000 persons living in Canada and Quebec, respectively. Fortunately, there is a high survival rate in childhood cancer. In fact, according to the Cancer in Young People in Canada (CYP-C) database, between 2012 and 2016, for all cancers combined, the one-year and five-year survival rates were 93.2% and 84.4%, respectively [3]. Treatment advances, better diagnostic accuracy and tools, participation in clinical trials, and treatment in specialized centers have all contributed to better survival rates in children [4,5]. However, pediatric cancer can cause emotional and financial problems for the child and their family during and after cancer treatment. Children who survive are also at risk of chronic or late-occurring effects such as endocrine complications or cardiomyopathy caused by the cancer therapy and the disease itself [5]. 

Despite many epidemiological studies, childhood cancer risk factors and etiology are yet to be fully understood. In previous studies, ionizing radiation in high doses was considered an important risk factor for central nervous system tumors and leukemia [6,7]. Some data also showed a link between childhood cancer and genetic predispositions or the exposition of some chemotherapy agents [8,9]. Finally, socioeconomic status may also play an etiologic role in childhood leukemia, according to some studies [10]. 

Thus, pediatric cancer has a significant importance for public health because it has multiple consequences on patients, their families, and the survivors as well. It also represents an important challenge since its causes are still mostly unknown. Detecting some spatial clusters could help to better identify potential underlying risk factors for childhood cancer. Therefore, spatial epidemiological studies are essential to find the most effective intervention or prevention methods in pediatric cancer and to guide clinicians in the management of children diagnosed with cancer. 

The information received and analyzed from the previous CYP-C project by Larouche et al. regarding the epidemiology of pediatric brain tumors in Canada showed the incidence discrepancy of central nervous system (CNS) tumors between some geographical areas in Quebec province [11]. Indeed, for patients diagnosed with CNS tumors in our province, the following five regions presented a higher age-adjusted incidence rate (AAIR): Chaudières-Appalaches (5.95), Mauricie and Centre-Du-Québec (4.22), Capitale-Nationale (4.18) and Outaouais (4.10). However, Chaudière-Appalaches is the only region that showed a significantly higher AAIR [11].

To our knowledge, there are no recent studies that were conducted on the geographical variation in all pediatric cancer incidence in the province of Quebec. If we confirm in this study that there are some areas where the incidence of tumors is higher, we could promote the development of further health research to have a greater understanding of risk factors, such as environment or genetic predisposition. Furthermore, it may ultimately help public health authorities to take specific actions in these areas [12].

The goal of this study is to provide descriptive statistics on the incidence rates (IRs) for all pediatric cancers in the population of Quebec from 2001 to 2018. We wanted to investigate the existence of statistically significant spatial-temporal clusters at the local, sub-regional, and regional levels and identify areas where the number of cases per child at risk is higher than in the province of Quebec.

## 3. Materials and Methods

### 3.1. Patients 

Patients’ data were extracted from the Cancer in Young People in Canada (CYP-C) surveillance system. CYP-C is a national population-based registry that collects information about pediatric cancer and benign CNS tumors, such as treatments, outcomes, and complications. We analyzed pediatric patients less than 15 years of age who were diagnosed between 2001 and 2018 with cancer in the province of Quebec. The following elements were analyzed during this study: age at diagnosis, sex, ethnicity, zip code, and type of cancer. Patients who were not living in the province of Quebec were excluded. Patients were divided into three groups based on their age at diagnosis: 0–4 years, 5–9 years, and 10–14 years. Cancer types were classified into three subgroups: leukemia/lymphoma, CNS tumors, and solid tumors. All types of cancer are described in the International Classification of Childhood Cancer, 3rd Edition (ICCC-3) [13]. In our study, the presence of cancer predisposition syndromes was unfortunately not known in our group of patients because these data were not formally included during the period 2001–2018 in the CYP-C database. This study was approved by the institutional Ethics Review Board and the CYP-C Management Committee. 

### 3.2. Geographical Data 

For the geographical analysis, three periods of diagnosis were established during the 17-year period analyzed: 2001–2006, 2007–2012, and 2013–2018. The place of residence of each patient was determined using the zip code at the time of diagnosis in the CYP-C database. To better assess regional variability in the incidence of pediatric cancer, we analyzed the cases of pediatric cancers according to the Quebec Health regions and local area health networks. 

### 3.3. Statistics 

To estimate the age-standardized incidence rate of pediatric tumors, supplementary information about the total number of children of the corresponding health region, year, gender, and age groups was obtained from population estimates and projections by health territory from the Institut de la Statistique du Québec based on the Canadian census data of 2001, 2006, and 2011. The incidence estimations of the pediatric tumors are presented graphically by health territory, and their evolution over time is also presented. The residence of each patient was estimated using the postal code of the residence at the time of diagnosis in the database. The postcodes were geocoded using the Postal Code Conversion File Plus (PCCF+). Geospatial data manipulation and mapping were performed using the ArcGIS Pro 2.6 software and R. 

Sociodemographic and clinical characteristics were described as categorical variables using frequencies and percentages. Poisson generalized estimating equation models, which were fitted to estimate the crude and adjusted incidence rates ratio. In the case of overdispersion problems, negative binomial models were applied. The Statistics Canada census data and the Institut de la Statistique du Québec estimations were used to obtain total population numbers as denominators detailed by postal code, sex, and age groups (0–4.99; 5–9.99; 10–14.99) for corresponding periods (2001–2005, 2006–2010, 2011–2018). The distribution of children’s age in 2001 was used for age-standardized incidence rate estimations in all models estimating the incidence rates. Statistical analyses will be performed using SAS Statistical Software v.9.4 (SAS Institute, Cary, NC, USA) with a two-sided significance level set at *p* < 0.05.

## 4. Results

### 4.1. Clinical Characteristics of the Patients

The population of Quebec at the beginning of the study period (2001) was 7,396,456 and 8,401,738 at the end of the study period (2018), including the group less than 15 years of age, corresponding to 0.18% and 0.16%, respectively. A total of 3904 pediatric patients living in the province of Quebec with a diagnosis of cancer or benign CNS tumors were identified between 2001 and 2018. There were 2096 males (53.7%) and 1808 females (46.3%), corresponding to a male/female ratio of 1.2:1. The majority (72.1%) of the participants were Caucasian. The proportion of children in the group age category was as follows: 47.8% in the group of 0–4 years, 25.9% in the group of 5–9 years, and 26.3% in the group of 10–15 years. IR for the age group 0–4 years was 25.0 per 100,000 person year, with a statistically significant difference compared to the IRs for the other age groups (*p* < 0.0001). In comparison, the overall IR in the province of Quebec was 16.14 (95%CL [15.56–16.73]) per 100,000 person years. The patient’s characteristics are summarized in Table 1.

### 4.2. Characteristics of the Cancers 

In the group of 3904 patients, 1662 were diagnosed with leukemia or lymphoma (42.6%), 928 with a CNS tumor (23.8%), and 1314 (33.6%) with a solid tumor. Overall, 25.9% of our patients had metastasis (n = 1012). The classification of tumors and IRs is also described in Table 1. 

Between 2001 and 2018, there was no statistically significant difference in the overall incidence of childhood cancers in the province of Quebec (Table 1).

### 4.3. Geographic Distribution in Quebec 

**Figure 1:**
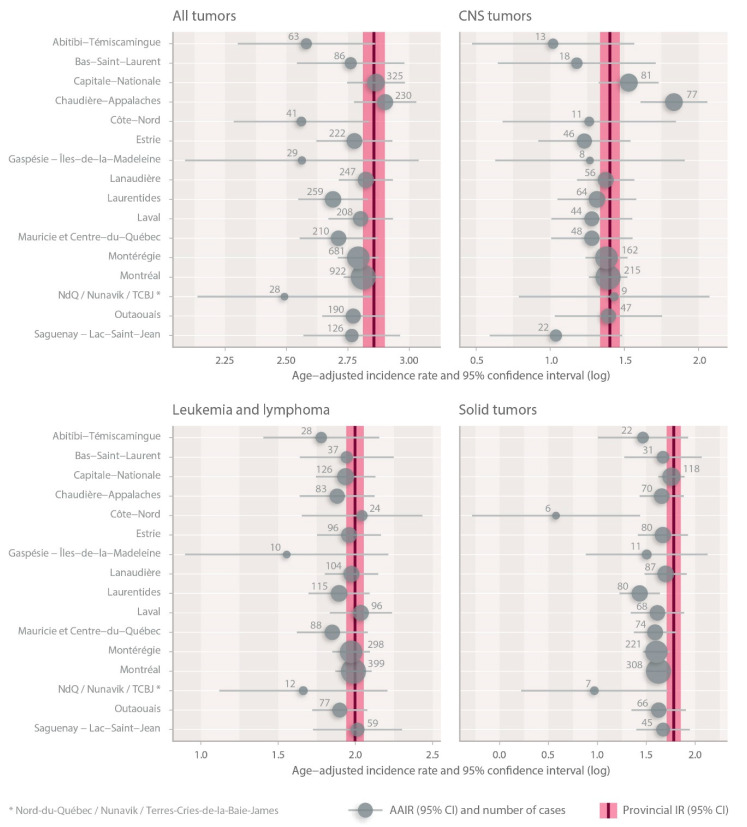
Incidence rate according to childhood cancer type (aged < 15 years) and Quebec Health regions during the period 2001–2018.

We calculated the age-adjusted incidence rate (AAIR) for each Quebec Health Region. Figure 1 represents the age-adjusted incidence rate according to childhood cancer types and health regions between 2001 and 2018 in the province of Quebec. In our cohort, for all childhood cancers, the five regions that presented a higher AAIR were Chaudière-Appalaches (18.2), Capitale-Nationale (17.5), Lanaudière (16.8), Montreal (16.6), and Laval (16.5). 

Also, between 2001 and 2018, for all childhood cancers, the AAIR in the areas of Chaudière-Appalaches was 1.19 (95%CL [1.04–1.35], *p* = 0.0090) times higher than the average AAIR in the province of Quebec. The same comparison shows that the AAIR in the Capitale-Nationale was 1.14 (95%CL [1.01–1.29], *p* = 0.0310) times higher than the provincial average AAIR.

**Figure 2:**
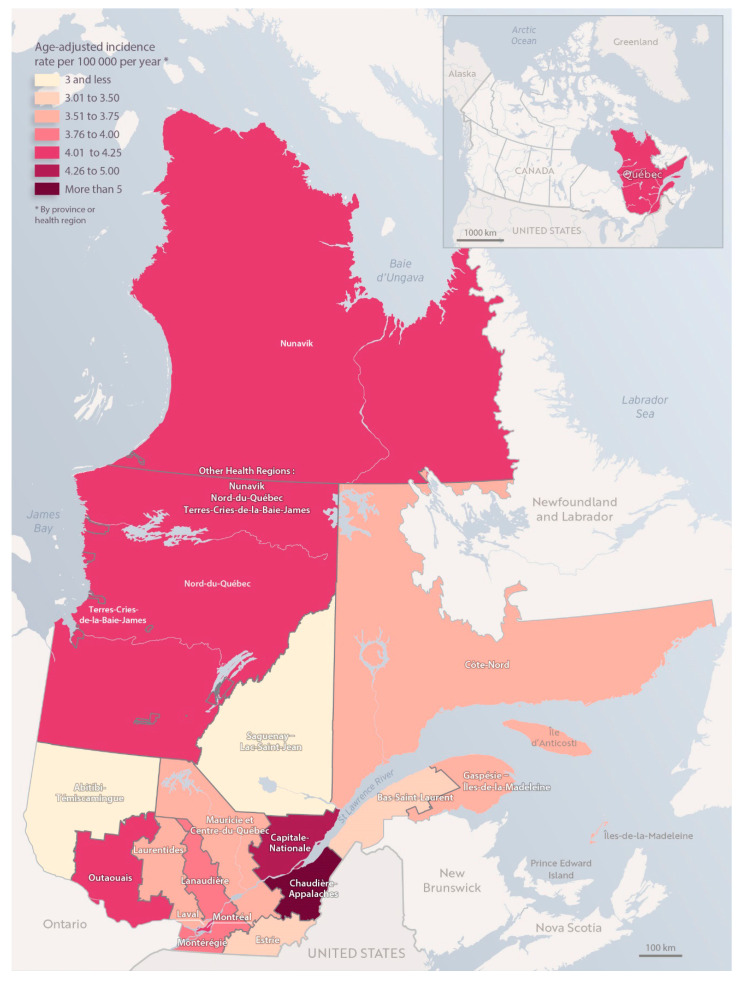
Map of the incidence rate of CNS tumors among children (aged < 15 years) in the province of Quebec, 2001–2018.

For the group of CNS tumors, when we looked at each Quebec Health Region, the AAIR was higher in Chaudière-Appalaches and Capitale-Nationale with rates of 6.3 and 4.6 per 100,000 person years, respectively (Figure 2). When comparing the AAIR of CNS tumors in Chaudière-Appalaches and in Capitale-Nationale with the provincial average, we noticed a statistically higher incidence in Chaudière-Appalaches and Capitale-Nationale (*p* < 0.0001 and *p* = 0.0602, respectively), as well.

For the group of solid tumors, the incidence in Capitale-Nationale was statistically higher than the IR in the province of Quebec (*p* = 0.0020). On the other hand, in the area of Côte-Nord, we saw a statistically significant decrease in AAIR (1.8 per 100,000 person years) when compared to the provincial AAIR (*p* = 0.0243), meaning that there could be fewer cases of solid tumors in this region. 

There was no statistically significant difference in the incidence of leukemia/lymphoma in each Quebec Health Region compared with the IR in the province of Quebec. 

## 5. Discussion 

The goal of this retrospective study was to provide statistics on the IRs of all childhood cancers among the Quebec population from 2001 to 2018 and to find statistically significant spatial-temporal clusters at regional levels. In our cohort, the population was mainly Caucasian, and the incidence rates were higher in males and in patients aged 0–4 years. These findings are also reported in the study of Siegel et al., where the CDC analyzed data from United States Cancer Statistics (USCS) over the period 2003 to 2014. Incidence rates for 0–4, 5–9, and 10–14 years were 22.9, 12.3, and 13.3 per 100,000 person years, respectively [12]. 

The childhood cancer group with the highest incidence rate was leukemia/lymphoma (6.99), as described in the literature, where leukemia was the most common childhood cancer, followed by CNS tumor and lymphoma [12,14,15].

The overall incidence of childhood cancers in Quebec between 2001 and 2018 was stable in our study. A large US study also reported, for the period 2001–2009, a stable incidence in the population younger than 20 years for leukemia, lymphoma, and CNS tumors [16]. By contrast, in a study by Xie et al., where the Canadian Cancer Registry (CCR) was used, for males and females of less than 15 years of age, we reported an increase in incidence by 0.5% annually from 1992 to 2010, respectively, and an increase by 3.2% from 2004 to 2010 in Canada [15]. The same observation was made in a European population-based registry study where the incidence increased significantly by 0.54% per year in children aged 0 to 14 years for the period 1991–2010 [17]. Variability in studies could be explained by varying patterns across regions and by different intervals of monitoring in each study. 

More importantly, we found that the Health Regions of Chaudière-Appalaches and Capitale-Nationale showed a significantly higher AAIR when compared to the provincial average for all childhood tumors and CNS tumors. These results are consistent with what has been previously reported in the study by Larouche et al. [11]. However, in this previous study, the Health Region of Chaudière-Appalaches was the only one that had a significantly higher AAIR for CNS tumors during the period 2001–2015. For solid tumors, only Capitale-Nationale showed a significantly higher AAIR in our study. We also demonstrated a significantly lower AAIR for solid tumors in Côte-Nord. Nevertheless, the low number of events in this region must be taken into consideration, and we must be careful in interpreting these data. To our knowledge, there are no other studies that have made these important findings in the province of Quebec. 

However, similar studies on the epidemiologic mapping of childhood cancers have been performed in Canada and other countries with reported evidence of geographic variation as well [18,19,20]. For example, a study by Holmes Jr. et al. on the epidemiologic, racial, and healthographic mapping of Delaware pediatric cancer between 2004 and 2014 described a significant childhood cancer variation for two zip codes (Newport and Lincoln). Newport is related to chemical companies and automotive industries, which could be associated with potential carcinogens. Lincoln is a community based on agriculture, and exposure to pesticides has been suggested consequently [19]. Indeed, the literature proposed that cancers may be explained by several factors, such as exposition to carcinogenic chemicals (e.g., drinking water, air pollution, environmental toxins) [21] and heritable genetic factors for a minority of cancers [22]. Cluster findings by the Centers for Disease and Prevention have suggested that infections could be associated with the development of leukemias and lymphomas, as well [23]. Finally, an association between exposition to metals and hepatocellular carcinoma or between lead and the Wilms tumor has been reported in some studies [24].

With these data, we can wonder whether there would be genetic and/or environmental issues that could explain the geographic variations in Chaudière-Appalaches and Capitale-Nationale. The Health Region of Chaudière-Appalaches is composed mostly of agriculture (20% of farms in the province of Quebec and 11% of cultivated areas) and industries (e.g., plastic, metallurgy, textiles). High numbers of soilless farms in Chaudière-Appalaches, where there are often pigs and poultry, can create a significant deterioration in the quality of surface water and potentially groundwater. In groundwater, there are sometimes high levels of arsenic [25]. Although most studies did not find an association between arsenic exposure and childhood cancers [26], a study by Liaw et al. suggested that exposure to high arsenic levels in drinking water may increase childhood liver cancer mortality [27]. Moreover, studies in the literature are also related to early-life exposure to arsenic and cancer development later in life [28]. The aqueduct network in Chaudière-Appalaches is composed in part of horizontal drains placed under meadows or pastures. Therefore, there is a high risk of bacteriological contamination as well as nitrates [25]. This could have an impact on the incidence of childhood cancers, as the literature suggests that exposure to nitrate from drinking water may increase the risk of CNS tumors [29]. 

Capitale-Nationale is in south-central Quebec, to the north of the St. Lawrence River. In its southern part, there is mostly agriculture and urbanization. In its northern part, forestry and mining predominate. In Portneuf, the quality of groundwater was affected in areas of intensive potato cultivation, with a nitrate value that exceeded the standard value. [30]. In Shannon, a study was conducted by the Regional Director of Public Health for the National Capital Region because of a report of the Regroupement des Citoyens de Shannon (RCS) that noted an abnormal number of cancer cases, especially among the residents of the Courcelette sector. Several environmental issues have been raised, such as the contamination of groundwater by trichloroethylene (TCE). The exposure of TCE is recognized as a risk factor for non-Hodgkin’s lymphoma, brain, kidney, and liver cancer. However, it was difficult to evaluate the duration and intensity of TCE exposure on citizens, and the study could not conclude on the real causality of the risk of cancer [31]. A judgment of Quebec Court Appeal was in favor of the potential risk of health integrity from TCE exposure and individual claims circulated last year for those who suffered harm arising from this [32].

Other examples of environmental concerns come from the use of pesticides. Public Health from the Monteregie area undertook a study to estimate the exposition of pesticides used for apple cultures on farmers, their children, and the population living in this area. Without surprise, these people had a higher urine concentration of pesticides 24 h and 7 days after pesticide pulverization [33]. In France, an ongoing study named Pestiriv is evaluating the exposition of pesticides used in vineyards on residents living there, and the distance in relation to this between homes and vines [34]. Also, in France, a recent publication demonstrated evidence of a slight increase in the risk of acute lymphoblastic leukemia in children living in an area of high viticulture density and the authors pointed out the hypothesis of environmental exposure to pesticides [35].

Thus, in the area described in our study, there might be an exposition of potential carcinogens around the environment that should be investigated, taking into consideration the impact of cancer on pediatric patients.

This study has several limitations. First, each Health Region is associated with a large area. Even if we found a geographical cluster in Chaudière-Appalaches, this territory remains very large, which may have limited our analysis. The use of CLSC territories could have been an alternative because they correspond to smaller areas. However, we attempted to estimate the incidence rate of childhood cancer based on local area health networks, and the number per network was too low, which could have affected the power of this study. Secondly, we had to combine the northern territories (Northern Quebec, Nunavik, Terre-Cries-Baie-James) for data analysis because there were too few events per region. Thirdly, in our study, the cancer predisposition syndrome status was unknown. Patients’ genetic assessment data were not formally included in the first version of the registry, and this restricted our ability to consider this variable in the geographical variation for childhood cancer incidence. 

## 6. Conclusions

In conclusion, this study reports statistics on the IRs of all childhood cancers for patients less than 15 years of age over a 17-year period. It suggests a significantly higher age-adjusted incidence of childhood cancers in certain geographic areas in the province of Quebec. Further research is needed to examine these findings and identify epidemiologic features that may help to find the underlying etiologies for the higher incidence of childhood cancers in these areas. 

## Figures and Tables

**Table 1:** Patient characteristics and age-standardized age-adjusted incidence rates of cancer diagnosed among children (aged < 15 years) in the province of Quebec, 2001–2018.

		Number (%)	AAIR/100,000 Person Years [95%CL]
All children		3904 (100%)	17.3 [16.6–18.2]
Age groups, n (%)	0–4 years	1867 (47.8)	25.0 [23.8–26.3]
	5–9 years	1011 (25.9)	13.2 [12.3–14.1]
	10–14 years	1026 (26.3)	12.9 [12.0–13.8]
Gender, n (%)	Male	2096 (53.7)	17.3 [16.4–18.3]
	Female	1808 (46.3)	15.0 [14.2–15.9]
Years, n (%)	2001–2006	1254 (32.1)	15.7 [14.8–16.7]
	2007–2012	1276 (32.7)	16.3 [15.4–17.3]
	2013–2018	1374 (35.2)	16.5 [15.5–17.6]
Classification of tumors, *n* (%)	Leukemia/lymphoma	1662 (42.6)	6.99 [6.64–7.36]
	Solid tumors	1314 (33.6)	5.01 [4.70–5.33]
	CNS tumors	928 (23.8)	3.97 [3.72–4.24]

## Data Availability

As per CYP-C policies, individual participant data will not be shared. Application for utilization of data can be submitted through the C17 Council website www.C17.ca/administration/Council (accessed on 12 March 2024).

## References

[1] Kadan-Lottick N.S., Kliegman R.M., Behrman R.E., Jenson H.B. (2007). Epidemiology of childhood and adolescent cancer. Nelson Textbook of Pediatrics.

[2] (2021). Leading Causes of Death, Total Population, by Age Group and Sex, Canada. https://www150.statcan.gc.ca/t1/tbl1/en/tv.action?pid=1310039401.

[3] Cancer in Young People in Canada Data Tool 2020 Edition. https://health-infobase.canada.ca/data-tools/cypc/.

[4] Canadian Cancer Society and the National Cancer Institute of Canada Canadian Cancer Statistics 2008. https://publications.gc.ca/collections/collection_2008/statcan/CS2-37-2008E.pdf.

[5] Eiser C., Absolom K., Greenfield D., Snowden J., Coleman R., Hancock B., Davies H. (2007). Late Effects Group, Sheffield (LEGS). Follow-up care for young adult survivors of cancer: Lessons from pediatrics. J. Cancer Surviv..

[6] Wiemels J. (2015). New insights into childhood leukemia etiology. Eur. J. Epidemiol..

[7] Wakeford R. (2013). The risk of childhood leukaemia following exposure to ionising radiation—A review. J. Radiol. Prot..

[8] Strahm B., Malkin D. (2006). Hereditary cancer predisposition in children: Genetic basis and clinical implications. Int. J. Cancer.

[9] Davies S.M. (2007). Subsequent malignant neoplasms in survivors of childhood cancer: Childhood Cancer Survivor Study (CCSS) studies. Pediatr. Blood Cancer.

[10] Borugian M.J., Spinelli J.J., Mezei G., Wilkins R., Abanto Z., McBride M.L. (2005). Childhood leukemia and socioeconomic status in Canada. Epidemiology.

[11] Larouche V., Toupin A.K., Lalonde B., Simonyan D., Jabado N., Perreault S. (2020). Incidence trends in pediatric central nervous system tumors in Canada: A 15 years report from Cancer and Young People in Canada (CYP-C) registry. Neurooncol. Adv..

[12] Siegel D.A., Li J., Henley S.J., Wilson R.J., Lunsford N.B., Van Dyne E.A. (2018). Geographic Variation in Pediatric Cancer Incidence—United States, 2003–2014. MMWR Morb. Mortal. Wkly. Rep..

[13] Fritz A., Percy C., Jack A., Shanmugaratnam K., Sobin L., Parkin D.M., Whelan S. (2000). International Classification of Diseases for Oncology.

[14] Li J., Thompson T.D., Miller J.W., Pollack L.A., Stewart S.L. (2008). Cancer incidence among children and adolescents in the United States, 2001–2003. Pediatrics.

[15] Xie L., Onysko J., Morrison H. (2018). Childhood cancer incidence in Canada: Demographic and geographic variation of temporal trends (1992–2010). Health Promot. Chronic Dis. Prev. Can..

[16] Siegel D.A., King J., Tai E., Buchanan N., Ajani U.A., Li J. (2014). Cancer incidence rates and trends among children and adolescents in the United States, 2001–2009. Pediatrics.

[17] Steliarova-Foucher E., Fidler M.M., Colombet M., Lacour B., Kaatsch P., Piñeros M., Soerjomataram I., Bray F., Coebergh J.W., Peris-Bonet R. (2018). Changing geographical patterns and trends in cancer incidence in children and adolescents in Europe, 1991–2010 (Automated Childhood Cancer Information System): A population-based study. Lancet Oncol..

[18] Amin R., Bohnert A., Holmes L., Rajasekaran A., Assanasen C. (2010). Epidemiologic mapping of Florida childhood cancer clusters. Pediatr. Blood Cancer.

[19] Holmes L., Vandenberg J., McClarin L., Dabney K. (2016). Epidemiologic, Racial and Healthographic Mapping of Delaware Pediatric Cancer: 2004–2014. Int. J. Environ. Res. Public Health.

[20] Torabi M., Singh H., Galloway K., Israels S.J. (2015). Geographical variation in the incidence of childhood leukaemia in Manitoba. J. Paediatr. Child Health.

[21] Carpenter D.O., Bushkin-Bedient S. (2013). Exposure to chemicals and radiation during childhood and risk for cancer later in life. J. Adolesc. Health.

[22] Lichtenstein P., Holm N.V., Verkasalo P.K., Iliadou A., Kaprio J., Koskenvuo M., Pukkala E., Skytthe A., Hemminki K. (2000). Environmental and heritable factors in the causation of cancer—Analyses of cohorts of twins from Sweden, Denmark, and Finland. N. Engl. J. Med..

[23] Heath C.W. (2005). Community clusters of childhood leukemia and lymphoma: Evidence of infection?. Am. J. Epidemiol..

[24] Ross J.A., Spector L.G., Schottenfeld D., Fraumeni J.F. (2006). Cancers in children. Cancer Epidemiology and Prevention.

[25] (2023). Regional Portrait of Water in Chaudière-Appalaches. https://www.environnement.gouv.qc.ca/eau/regions/region12/12-chaudiere(suite).htm#61.

[26] Engel A., Lamm S.H. (2008). Arsenic exposure and childhood cancer—A systematic review of the literature. J. Environ. Health.

[27] Liaw J., Marshall G., Yuan Y., Ferreccio C., Steinmaus C., Smith A.H. (2008). Increased childhood liver cancer mortality and arsenic in drinking water in northern Chile. Cancer Epidemiol. Biomarkers Prev..

[28] Roh T., Steinmaus C., Marshall G., Ferreccio C., Liaw J., Smith A.H. (2019). Age at Exposure to Arsenic in Water and Mortality 30–40 Years After Exposure Cessation. Am. J. Epidemiol..

[29] Stayner L.T., Schullehner J., Semark B.D., Jensen A.S., Trabjerg B.B., Pedersen M., Olsen J., Hansen B., Ward M.H., Jones R.R. (2021). Exposure to nitrate from drinking water and the risk of childhood cancer in Denmark. Environ. Int..

[30] (2023). Regional Portrait of Water in Capitale-Nationale. https://www.environnement.gouv.qc.ca/eau/regions/region03/03-capitale(suite).htm#7.

[31] (2016). Study of the Incidence of Brain Cancer Cases, Kidney, Liver, and Non-Hodgkin Lymphoma among People Who Lived in the Municipality of Shannon. https://www.ciusss-capitalenationale.gouv.qc.ca/node/2685.

[32] (2021). Contamination of Groundwater by TCE in Shannon, Quebec. Spieser V. Attorney General of Canada et Al. Raymond Chabot Grant Thornton. https://shannonactioncollective.ca/smartlets/do.aspx?interviewID=home&workspace=claims-shannon&lang=en.

[33] Moreau P. (1999). Étude Exploratoire sur l’Évaluation de l’Impact de l’Utilisation des Organophosphorés sur la Santé de la Population Limitrophe aux Vergers de la Montérégie. https://savoirs.usherbrooke.ca/handle/11143/381.

[34] PestiRiv: Une Étude pour Mieux Connaître l’Exposition aux Pesticides des Personnes Vivant en Zones Viticoles et Non-Viticoles. https://www.santepubliquefrance.fr/etudes-et-enquetes/pestiriv-une-etude-pour-mieux-connaitre-l-exposition-aux-pesticides-des-personnes-vivant-en-zones-viticoles-et-non-viticoles.

[35] Mancini M., Hémon D., de Crouy-Chanel P., Guldner L., Faure L., Clavel J., Goujon S. (2023). Association between residential proximity to viticultural areas and childhood acute lymphoblastic leukemia risk in Mainland France: GEOCAP Case-Control study 2006–2013. Environ. Health Perspect..

